# Supplementary figures and images for: Locally invasive, castrate-resistant prostate cancer in a Pten/Trp53 double knockout mouse model of prostate cancer monitored with non-invasive bioluminescent imaging
Source: PLoS One. 2020 Sep 28;15(9):e0232807. doi: 10.1371/journal.pone.0232807 (PMC7521703; doi:10.1371/journal.pone.0232807)

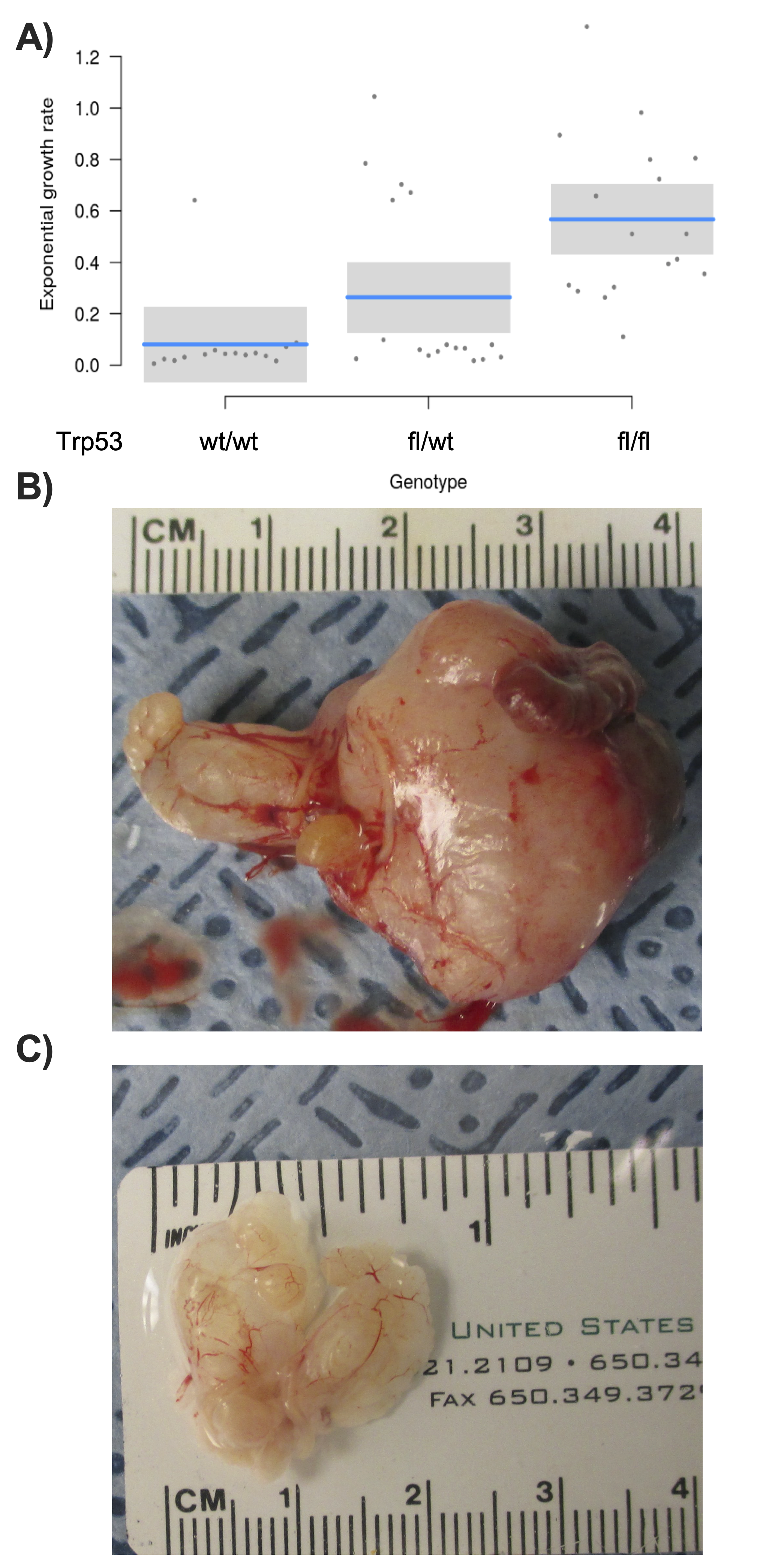

Supplement: S1 Fig — (A) Graph of the exponential growth rate for each mouse from the Pten fl/fl; Trp53: wt/wt, fl/wt, and fl/fl groups. Blue line is the mean with grey boxes outlining the 95% confidence intervals. (B) Gross anatomical image of prostate tumor from a DKO mouse at endpoint (approximately 25 weeks). A blood-filled seminal vesicle is evident at the top right of the image. (C) Gross anatomical image of prostate from WT (Pten only knockout) mouse at approximately 25 weeks. Note in this specimen seminal vesicles are readily apparent. (TIF) [file pone.0232807.s001.tif]

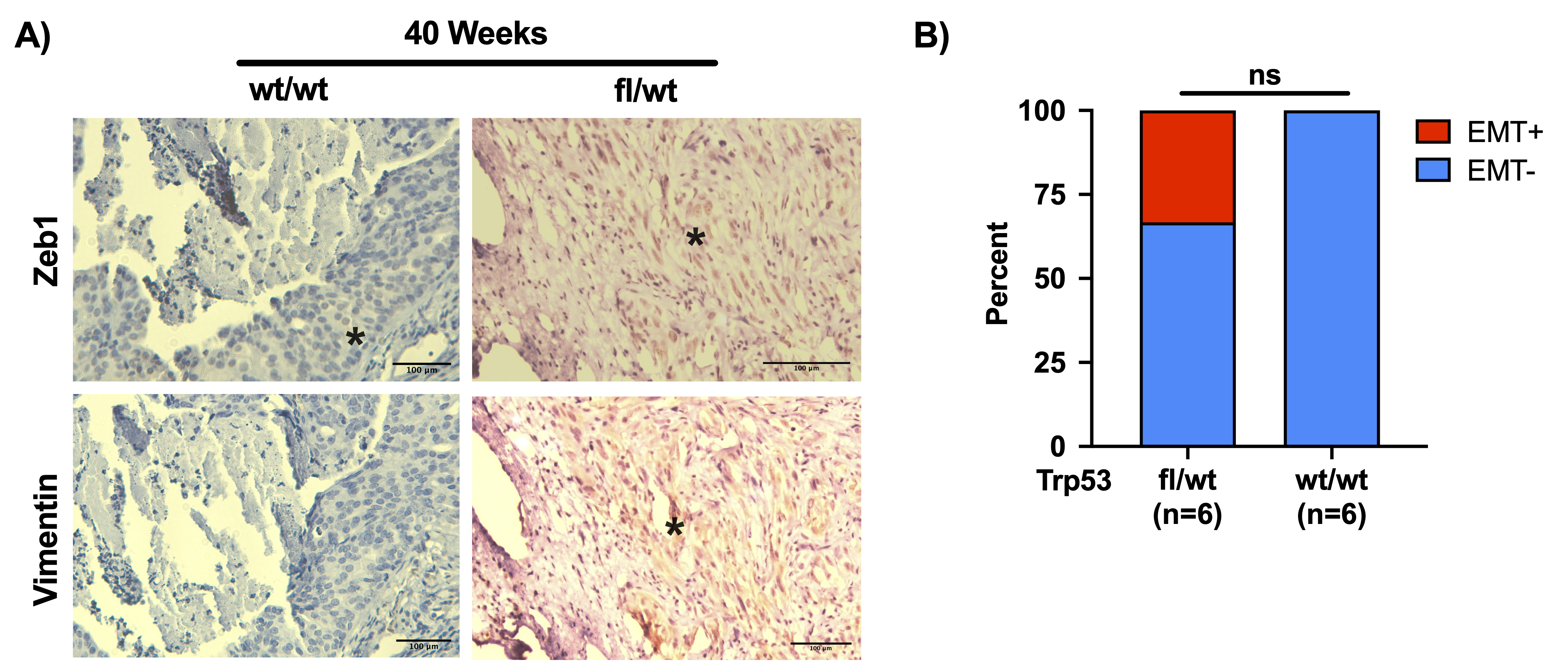

Supplement: S2 Fig — (A) Images of sections stained for Zeb1 and vimentin in WT and HET mice at 40 weeks, asterisk designates regions of positivity. Bar = 100 μm (B) Analysis of EMT status in these mice at this time point (p>0.05, Chi-Square test). (TIF) [file pone.0232807.s002.tif]

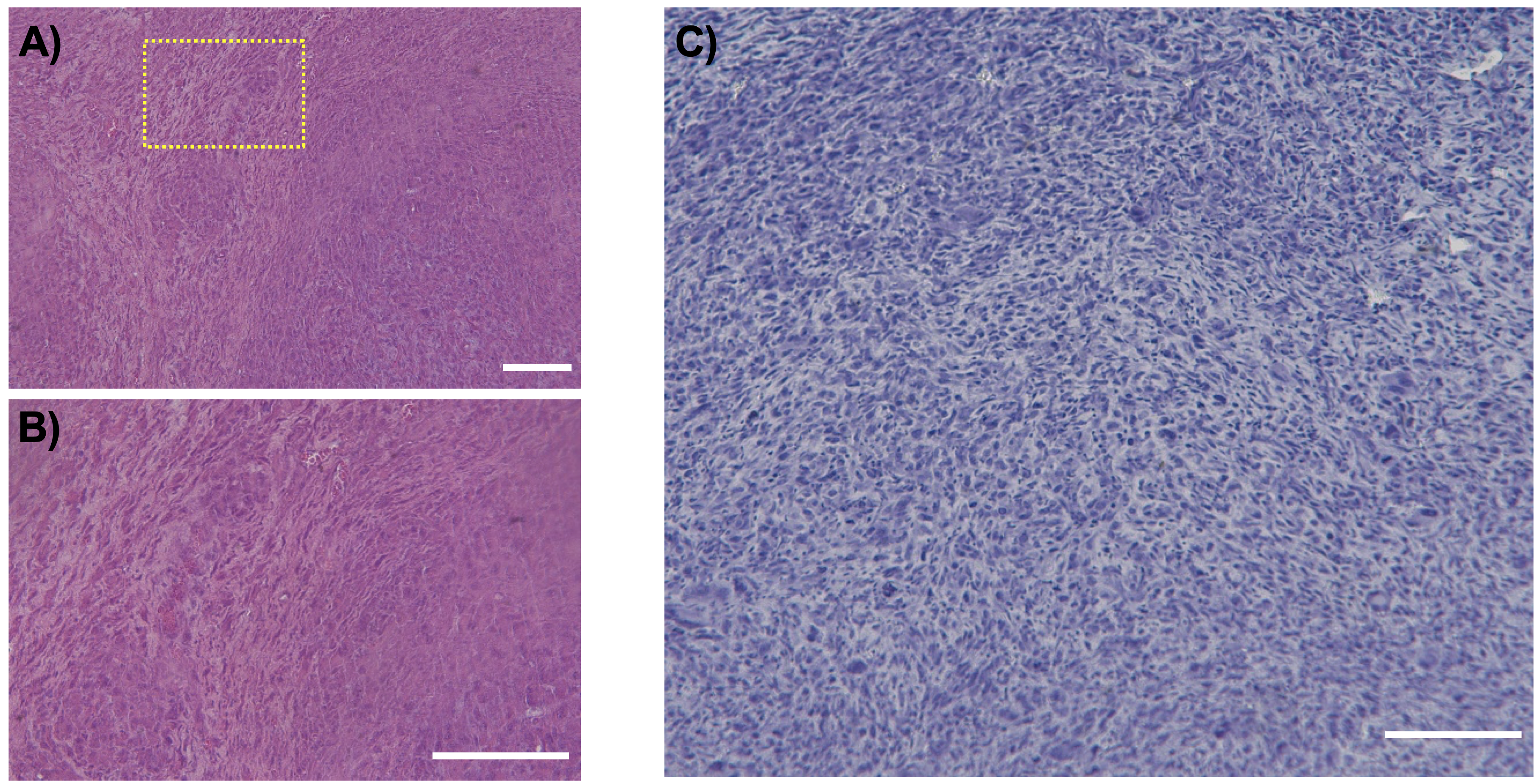

Supplement: S3 Fig — (A, B) H&E stain showing sarcomatoid carcinoma, similar to the non-castrated DKO mice. Yellow box in (A) is enlarged in (B) to show sarcomatoid architecture. (C) Immunostaining for AR. Bar = 150 μm. (TIF) [file pone.0232807.s003.tif]
